# Diallel Analysis of Early Leaf Spot (*Cercospora arachidicola* Hori) Disease Resistance in Groundnut

**DOI:** 10.3390/agronomy9010015

**Published:** 2019-01-01

**Authors:** Adama Zongo, Abdourasmane K. Konate, Kadidia Koïta, Mahamadou Sawadogo, Philippe Sankara, Bonny R. Ntare, Haile Desmae

**Affiliations:** 1 Environmental and Rural Development Sciences Institute (ERDSI), University of Dedougou, Dedougou BP 176, Burkina Faso; zongoadama87@gmail.com; 2 Institut de l’Environnement et de la Recherche Agricole (INERA), Bobo Dioulasso BP 910, Burkina Faso; kadougoudiou@gmail.com; 3 Life and Earth Sciences Faculty, University Ouaga I Pr. Joseph Ki-Zerbo, Ouagadougou BP 7021, Burkina Faso; benbakady@yahoo.fr (K.K.); sawadogomahamadou@yahoo.fr (M.S.); philippe.sankara@idr.fr (P.S.); 4 International Crops Research Institute for the Semi-Arid Tropics (ICRISAT), Bamako BP 320, Mali; bnatre@gmail.com

**Keywords:** general combining ability, specific combining ability, diallel, early leaf spot, groundnut

## Abstract

Early leaf spot (ELS) is one of the major biotic constraints of groundnut production in West and Central Africa. A study using 6 × 6 F2 full diallel populations from six parents (NAMA, B188, PC79-79, QH243C, TS32-1, and CN94C) was conducted to assess the mode of inheritance of ELS resistance traits. The F2 and parents were grown in a randomized complete block design with three replications. Data was collected on ELS disease severity, and an area under disease progress curve (AUDPC) was estimated. The results revealed that additive and non-additive gene actions were involved in the inheritance of the ELS resistance traits, but additive gene action was predominant. Significant reciprocal cross effect was observed, suggesting cytoplasmic effect on ELS resistance. Graphical analysis also revealed the predominance of additive gene action for ELS resistance. The results suggest that early generation selection should be effective for ELS resistance. Looking at the distribution of array points along with the regression line, parental lines NAMA, PC79-79, and B188 would be suitable as good donors in an ELS disease resistance breeding program.

## 1. Introduction

Groundnut or peanut (*Arachis hypogaea* L.) is an important oilseed crop in the world. It is a key crop for small farmers, especially in Africa and Asia, chiefly cultivated for its oil and food value. It contains about 45–56% of high quality edible oil, 25–30% protein, 20% carbohydrate, and is a rich source of dietary fiber, minerals, and vitamins E and B [1,2]. The haulm is a nutritious animal feed. In addition, groundnut helps to maintain soil fertility by fixing atmospheric nitrogen to the soil.

In 2016, the global groundnut production was about 44 million (M) tons from an area covering about 27.7 M hectares. The continent of Asia ranked first with 60.1% of global production, followed by Africa with about 29% of global production. The largest groundnut producing countries were China and India with 16.7 M and 6.9 M tons, respectively. Nigeria ranked first and third in Africa and the world, respectively, with about 3 M tons in 2016. In Burkina Faso, groundnut production was estimated at about 335,715 tons in 2016, obtained from an area of about 420,000 hectares, and with an average yield of about 799.3 kg/ha [3]. In general, groundnut productivity in Africa had the lowest average yield (902.6 kg/ha) in 2016 compared to America (3381.4 kg/ha), Asia (2186.8 kg/ha), Oceania (1947.3 kg/ha), Europe (3102.1 kg/ha), and the global average yield (1590.1 kg/ha) [3]. Several abiotic, biotic, and socio-economic constraints are responsible for the low yield in Africa. Among biotic factors, foliar fungal diseases are the major production constraints of groundnut [1,2]. Of these, early leaf spot (ELS), caused by *Cercospora arachidicola*, is a major and widely distributed disease. This disease, combined with rust or late leaf spot, can cause from 50% to 70% yield losses [4].

Control methods have been developed to minimize yield losses due to ELS, which include host plant resistance, and cultural and chemical control methods [5]. Fungicides, such as Maneb and Mancozeb, which belong to the ethylene bisdithiocarbamate group of chemicals, are effective in controlling ELS. However, chemical controls may not be economical for smallholder farmers in developing countries due to a number of reasons. These include low basic yields; difficulties in obtaining fungicides and application machinery, and their high costs; the problem of access to sources of clean water and of transporting it in sufficient quantities for high- or medium-volume spraying; lack of expertise, and lack of advice on the use of spray machinery and on its maintenance; and low or fluctuating prices for groundnut, discouraging farmers from risk-taking investment in the crop [6]. Besides, chemicals are potentially harmful to the environment and its inhabitants. Therefore, host plant resistance is considered the cheapest and most effective control method. Accordingly, the development of high yielding cultivars with resistance to ELS is an important breeding priority to increase groundnut productivity and reduce the impacts of the disease. A nine-point disease scale is used for measuring reactions to ELS [6]. Although complete resistance to ELS has not been found in the cultivated groundnut, several promising lines were identified as resistance sources [7–9]. The search for new sources of ELS resistance both from cultivated and wild relatives is a continuous process to developing ELS-resistant and high yielding varieties that are adaptable to target environments and preferred by farmers, markets, and consumers.

Understanding the inheritance of ELS is important to breeding for resistant groundnut cultivars. Previous studies revealed that resistance to ELS is recessive and independently inherited, and both quantitative and qualitative inheritance have been reported [10]. Genetic analysis using diallel mating design is useful to identify genotypes with combining ability in a desirable direction, and to identify parents with additive and non-additive effects for specific traits that may be used in breeding [11–13]. The knowledge on combining the ability and type of gene action responsible for the regulation of expression of ELS disease helps in planning appropriate breeding strategies. Therefore, this study was set up to generate data on ELS inheritance for groundnut breeding programs in West and Central Africa using groundnut genotypes from the region. The specific objectives were: (1) elucidating the mode of inheritance of genes governing the expression of ELS disease in populations under this study, (2) identifying good general combiners to be used as donor parents for ELS disease resistance, and (3) evaluating the breeding potential of the populations to identify ELS-resistant varieties.

## 2. Materials and Methods

### 2.1 Experiment Site

A field experiment was conducted from July to October 2014 in the research station of the Institute of Rural Development (IDR) of Nazi Boni University. The station is located in Gampela (12°22ʹ N and 12°25ʺ E), 18 km from Ouagadougou in the central region of Burkina Faso. The region is within the Sudano-Sahelian agro-ecological zone. The climate is characterized by dry and wet seasons with an annual rainfall varying from 700 to 900 mm that is fairly distributed over the rainy (wet) season of June to October.

### 2.2. Plant Material

Six groundnut genotypes were selected, on the basis of resistance to ELS disease, as parents for the study. Three resistant genotypes (NAMA, PC79-79, and B188) and 3 susceptible genotypes (QH243C, TS32-1, and CN94C) were used for developing F1 populations by using diallel cross design. NAMA is a local cultivar grown in Burkina Faso, whereas PC79-79 and B188 are improved genotypes from the Institut Sénégalais de Recherches Agricoles (ISRA) in Senegal, and Texas in the USA, respectively. The susceptible parents QH243C, TS32-1, and CN94C were from the Institut de l’Environnement et de Recherches Agricoles (INERA) in Burkina Faso, as seen in Table 1.

The 6 selected parents were crossed in hybridization blocks at the International Crops Research Institute for the Semi-Arid Tropics (ICRISAT), located at Samanko, Mali, following a 6 × 6 full diallel mating design. The resulting F_1_ hybrid seeds were grown to produce F_2_ progenies through self pollination. These 30 F_2_ populations and their 6 parents were evaluated under natural ELS infestation conditions at Gampela in the central region of Burkina Faso.

**Table 1.** Parents used and their origin.

Parent	Genotypes	Origin	Botanical Type	Reaction to ELS	Source
1	NAMA	Local/Burkina Faso	Virginia bunch	R	[14–16]
2	B188	Texas/USA	Spanish bunch	R	[16]
3	PC79-79	Institut Sénégalais de Recherches Agricoles (ISRA)/Senegal	Spanish bunch	R	[15,16]
4	QH243C	Institut de l’Environnement et de Recherches Agricoles (INERA)/Burkina Faso	Spanish bunch	S	[17]
5	TS32-1	Institut de Recherches pour les Huiles et Oléagineux (IRHO)/Burkina Faso	Spanish bunch	S	[15–17]
6	CN94C	INERA/Burkina Faso	Spanish bunch	S	[15–17]

Early Leaf Spot (ELS): R = Resistant, S = Susceptible.

### 2.3. Experimental Design and Data Collection

The 36 genotypes (i.e., 30 F2s and 6 parents) were grown in a randomized complete block design, with 3 replications during the 2014 rainy season, for evaluation under natural ELS infestation conditions at the experimental station of Gampela, Burkina Faso. This experimental station is known to be a hotspot for leaf spot diseases, including ELS. Each entry was planted in a single row plot of 4 m length and 50 cm apart. Plants were spaced 20 cm apart within rows. Seeds were treated using a fungicide (APRON STAR 42 WS) before sowing. Recommended cultural practices were followed during the growing season. ELS incidence was scored on groups of plants at 40, 60, and 80 days after planting using the 9-point field scale of ICRISAT [18]. A score of 1 was given if there was 0% infection; 2 for 1–5%; 3 for 6–10%; 4 for 11–20%, 5 for 21–30%; 6 for 31–40%; 7 for 41–60%; 8 for 61–80%; and 9 for 81–100% infection. In all assessments, disease score was averaged for all plants in each row.

In addition, the area under disease progress curve (AUDPC) was estimated for each plot using the formula of Shaner and Finnery [19] and applied by Debele and Ayalew [20]. AUDPC was estimated using ELS severity scores at 40, 60, and 80 days after sowing.

$$AUDPC\;=\;\sum_{n=1}^{n-1}0.5\left[\left(x_{i}\;+\;x_{i+1}\right)\;\left(t_{i+1}\;-\;t_{i}\right)\right]$$ (1)

where, *n* = total number assessment times, *t*_i_ = time of the ith assessment in days from the first assessment date, *x*_i_ = percentage of disease severity at ith assessment. AUDPC was expressed in percent-days.

The AUDPC values and the last ELS severity score (80 days after sowing) were used for statistical analysis.

### 2.4 Statistical Analysis

The ELS severity scores and AUDPC estimates were first subjected to linear mixed model and Friedman’s nonparametric ANOVA analyses, using the Genstat 15th Edition software, to test the significance of the differences between genotypes. Then, genetic analysis was performed using DIAL98 software [21]. Griffing’s method 2 and model 1 [22] was used to analyze the general combining ability (GCA) of progenies and specific combining ability (SCA) of crosses, supplemented by analysis using Hayman’s approach [23,24]. Besides, the graphical approach of Hayman was applied to test the adequacy of the dominance–additive model, the degree of dominance, and the direction of the dominance.

## 3. Results

### 3.1 Analysis of Variance and Mean Performance of the Diallel Population

The linear mixed model and Friedman’s nonparametric ANOVA results for ELS severity and the AUDPC of the 6 × 6 F_2_ full diallel population are presented in Table 2. The results showed highly significant differences (*p* < 0.01) among parents and F_2_ populations for the disease severity score and AUDPC.

**Table 2.** Results of the linear mixed model and Friedman’s nonparametric ANOVA for early leaf spot (ELS) disease score and area under disease progress curve (AUDPC) in 6 × 6 F_2_ diallel populations.

Linear Mixed Model Analysis					
Source of Variation	Degree of Freedom	ELS Severity		AUDPC	
		Wald Statistic	*F* Value	Wald Statistic	*F* Value
Replication	2	3.42	1.71 Ns	1.92	0.96 Ns
Genotype	35	558.52	15.96 ^**^	778.44	22.24 ^**^
Residuals	70	0.281		12,630	
**Friedman’s Nonparametric ANOVA**					
**Parameters**	**Degree of Freedom**	**Critical Value (*N* ≥ 30)**	**ELS Severity**	**AUDPC**	
Friedman’s statistic	35	50.89	83.81 ^**^	93.27 ^**^	
Adjusted for ties	35	50.89	90.98 ^**^	96.06 ^**^	

Ns = not significant;

** significant at *p* < 0.01.

The mean performance for both traits and Friedman’s rank are presented in Table 3. Low ELS severity scores of 3 were recorded by parents B188, PC79-79, and NAMA, followed by crosses B188 × NAMA, NAMA × B188, and NAMA × PC79-79. In addition, crosses B188 × PC79-79, TS32-1 × PC79-79, PC79-79 × NAMA, CN94C × PC79-79, QH243C × NAMA, TS32-1 × NAMA, TS32-1 × B188, CN94C × NAMA, QH243C × PC79-79, PC79-79 × B188, and QH243 × B188 recorded a moderate ELS severity score of 5. A high disease severity score of 8 was obtained for parent TS32-1, followed by crosses B188 × QH243C, B188 × CN94C, B188 × TS32-1, PC79-79 × CN94C, CN94C × TS32-1, TS32-1 × CN94C, and parents CN94C and QH243C.

**Table 3.** Mean performance and Friedman’s rank of genotypes for ELS severity score and AUDPC.

Parents and Crosses	ELS Severity		AUDPC	
	ELS Score	Friedman’s Rank	AUDPC Value	Friedman’s Rank
NAMA	3	2.00	350	2.33
B188	3	2.00	400	3.17
PC79-79	3	2.00	400	3.17
PC79-79 × NAMA	5	13.30	433	4.17
PC79-79 × B188	5	16.70	433	4.17
B188 × NAMA	4	5.17	467	5.83
NAMA × B188	4	5.17	467	5.83
NAMA × PC79-79	4	7.67	600	8.67
NAMA × CN94C	6	24.20	600	9.17
B188 × PC79-79	5	12.30	700	12.33
NAMA × TS32-1	6	24.20	800	13.83
PC79-79 × QH243C	6	24.20	800	13.83
PC79-79 × TS32-1	6	24.20	800	13.83
B188 × TS32-1	7	30.30	800	13.83
B188 × QH243C	7	27.50	767	15.00
PC79-79 × CN94C	7	30.30	800	15.50
NAMA × QH243C	6	20.70	867	15.83
B188 × CN94C	7	27.50	867	17.83
QH243C × NAMA	5	12.30	933	19.33
QH243C × B188	5	15.70	933	20.17
TS32-1 × PC79-79	5	12.30	1033	22.67
QH243C × PC79-79	5	12.30	1033	23.67
TS32-1 × QH243C	6	20.70	1100	25.17
CN94C × QH243C	6	24.00	1100	25.17
CN94C × NAMA	5	12.30	1100	25.33
CN94C × PC79-79	5	12.30	1100	25.33
TS32-1 × NAMA	5	12.30	1100	25.33
TS32-1 × B188	5	12.30	1167	27.17
CN94C × B188	6	19.20	1167	27.83
QH243C × TS32-1	6	20.70	1167	28.00
QH243C	7	30.30	1233	30.67
TS32-1 × CN94C	7	30.30	1233	30.67
CN94C	7	30.30	1267	31.17
QH243C × CN94C	6	24.20	1300	32.50
CN94C × TS32-1	7	30.30	1300	32.50
TS32-1	8	34.70	1400	35.00

**Table 4.** Griffing’s ANOVA for the ELS severity score and AUDPC in 6 × 6 full diallel cross of groundnut.

Source of Variation	Degree of Freedom	ELS Severity		AUDPC	
		Mean Square	*F* Value	Mean Square	*F* Value
Replication	2	0.43	1.31 Ns	14782.5	1.06 Ns
General combining ability (GCA)	5	7.08	10.09 ^**^	862277.1	34.72 ^**^
Specific combining ability (SCA)	9	0.7	2.13 ^*^	24833.71	1.78 Ns
Reciprocal	15	1.94	5.89 ^**^	94333.34	6.75 ^**^
Error	58	0.33		13973.18	
Total	89				
**Variance Components**					
		**ELS Severity**		**AUDPC**	
GCA		0.295		35928.241	
SCA		0.117		4138.889	
GCA/SCA		2.521		8.681	

Ns = not significant;

* significant at *p* < 0.05;

** significant at *p* < 0.01.

AUDPC values ranged from 350 for parent NAMA to 1400 recorded for parent TS32-1. In general, parents NAMA, B188, and PC79-79 recorded the lowest AUDPC. Crosses PC79-79 × B188, PC79-79 × NAMA, NAMA × B188, B188 × NAMA, NAMA × CN94C, NAMA × PC79-79, B188 × PC79-79, and B188 × QH243C recorded moderate AUDPC values.

### 3.2. Diallel Analysis Using the Griffing Model

### 3.2.1 Griffing Analysis of Variance for ELS Resistance

Table 4 shows the analysis of variance of diallel crosses in genotypes for the ELS disease score and AUDPC according to the Griffing model. The results showed highly significant differences (*p* < 0.01) for the general combining ability (GCA) and a reciprocal effect for the ELS severity score and AUDPC, while specific combining ability (SCA) mean squares were significant for the ELS severity score and not significant for the AUDPC. In addition, the GCA and SCA variance components were significantly different to zero for the ELS severity score and AUDPC. The ratios of the GCA and SCA variances were significantly higher than unity, with values of 2.521 and 8.681 for ELS severity and AUDPC, respectively.

### 3.2.2 General and Specific Combining Ability Effects of the Crosses

The GCA represents the average performance of a line in a cross combination, whereas SCA represents those cases in which certain combinations do relatively better or worse than would be expected on the basis of the average performance of the lines involved [22]. The GCA and SCA estimates for parents and crosses are provided in Table 5. The Parents NAMA, B188, and PC79-79 showed negative GCA values for the ELS score and AUDPC, while the parents QH243C, TS32-1, and CN94C showed positive GCA for both traits. Of 30 crosses, negative SCAs were observed for both traits in five crosses, including NAMA × B188, PC79-79 × TS32-1, QH243C × TS32-1, PC79-79 × CN94C, and QH243C × CN94C. In addition, four other crosses, including NAMA × PC79-79, B188 × PC79-79, B188 × QH243C, and NAMA × CN94C, showed negative SCA values for AUDPC only.

**Table 5.** GCA (diagonal, bold) and SCA effects of parental lines and crosses for resistance to early leaf spot of groundnut.

Parents	ELS Severity					
	NAMA	B188	PC79-79	QH243C	TS32-1	CN94C
NAMA	−0.75	−0.66	0.05	0.30	0.26	0.05
B188		−0.29	0.09	0.34	0.13	0.09
PC79-79			−0.33	0.05	−0.16	−0.03
QH243C				0.25	−0.41	−0.28
TS32-1					0.46	0.18
CN94C						0.67
**AUDPC**						
NAMA	−202.78	−76.67	−22.50	77.50	65.00	−43.33
B188		−152.78	−22.50	−22.50	48.33	73.33
PC79-79			−156.94	48.33	−14.17	10.83
QH243C				126.39	−80.83	−22.33
TS32-1					188.89	−18.33
CN94C						197.22

### 3.3. Diallel Analysis Using the Hayman Model

### 3.3.1 Hayman Analysis of Variance for ELS Resistance

The Hayman analysis of variance for a 6 × 6 full diallel set of genotypes revealed that both additive “a” and dominance “b” components were highly significant for the ELS severity score and AUDPC, as seen in Table 6. The fractions of the dominance effects b1 (mean of dominance), b2 (symmetrical distribution of the alleles determining the dominance), and b3 (residual dominance effects) were significant for the ELS severity score, whereas only b2 was significant for the AUDPC. Maternal effect “c” and reciprocal effect “d” were significant for the AUDPC. However, the ELS severity score showed a significant effect only for maternal effect.

**Table 6.** Hayman ANOVA for the ELS severity score and AUDPC in a 6 × 6 full diallel cross of groundnut.

Source of Variation	Degree of Freedom	ELS Severity		AUDPC	
		Mean Square	*F* Value	Mean Square	*F* Value
Replications	2	0.48	1.71 Ns	12,105.00	0.96 Ns
a	5	19.42	68.99 ^**^	1590,826.00	125.95 ^**^
b	15	2.06	7.33 ^**^	30,853.24	2.44 ^**^
b1	1	5.2	18.48 ^**^	49,115.65	3.89 Ns
b2	5	3.89	13.81 ^**^	38,037.68	3.01 ^*^
b3	9	0.7	2.49 ^*^	24,832.73	1.97 Ns
c	5	5.56	19.74 ^**^	215,555.5	17.07 ^**^
d	10	0.14	0.49 Ns	33,722.26	2.67 ^**^
Error	70	0.28		12,630.29	
Total	107				

Ns = not significant;

* significant at *p* < 0.05;

** significant at *p* < 0.01.

### 3.3.2 Hayman Genetic Parameters for ELS Resistance

The estimates of genetic variation, based on Hayman’s approach, are shown in Table 7. The ELS severity score and AUDPC, respectively, had 5.314 and 251,317.7 additive variance values (D), 1.989 and 17,794.69 dominance variance 1 (H^1^), and 1.194 and 12,382.65 dominance variance 2 (H^2^). The magnitudes of the additive variances (D) were greater than the dominance variances (H^1^ and H^2^) for the two traits. Estimates of the distribution or relative frequency of dominant versus recessive genes (F), which measure the covariance of the additive and dominance effect, showed positive values for both traits. The average degree of dominance (H^1^/D)^1/2^ was 0.612 and 0.266 for ELS severity and AUDPC, respectively, which was less than unity for both traits. Estimates of heritability in a broad sense (Hbs%) and heritability in a narrow sense (Hns%) showed a high magnitude (>60%) for both traits, 93.5% and 73% for the ELS severity score, and of 95.6% and 92.3% for AUDPC, respectively.

**Table 7.** Estimates of the genetic parameters for the ELS severity score and AUDPC according to Hayman’s method in a 6 × 6 full diallel cross of groundnut.

Parameters	Description	ELS Severity (*±*SE)	AUDPC (*±*SE)
D	Additive variance	5.314 ± 0.6564	251,317.7 ± 30552.8
H^1^	Dominance variance 1	1.989 ± 0.4617	17,794.69 ± 11005.2
H^2^	Dominance variance 2	1.194 ± 0.2759	12,382.65 ± 7482.6
F	Product of additive by dominance	3.989 ± 0.7518	81,608.77 ± 28798.6
(H^1^/D)^1/2^	Average degree of dominance	0.612 ± 0.046	0.266 ± 0.056
kd/(kd + kr)	proportion of dominance genes	0.807 ± 0.0167	0.8051 ± 0.0514
Hbs %	Heritability for diallel in a broad sense	93.50 ± 0.011	95.60 ± 0.008
Hns %	Heritability for diallel in a narrow sense	73.00 ± 0.038	92.30 ± 0.019

### 3.3.3. Hayman Graphical Analysis

**Figure 1.**
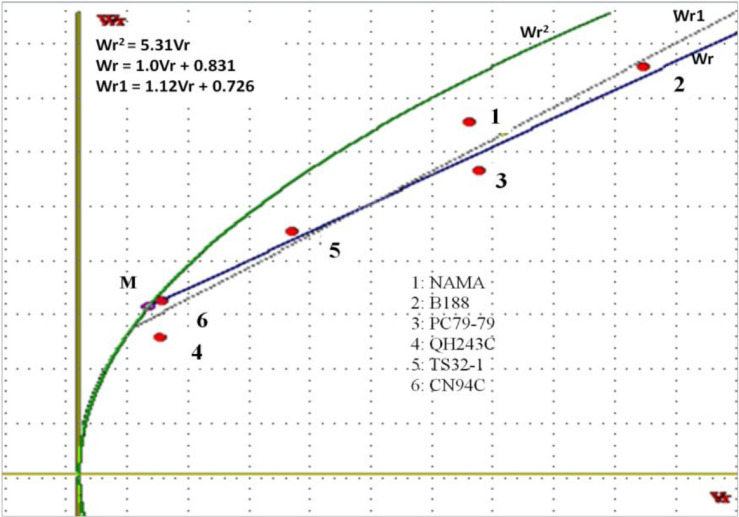
Covariance (Wr)/variance (Vr) graph for the ELS severity score of groundnut. Wr: covariance between a parent r and its progeny; Vr: variance between a parent r and its progeny; Wr1: regression line; Wr^2^: parabola; Wr: tangent to parabola.

**Figure 2.**
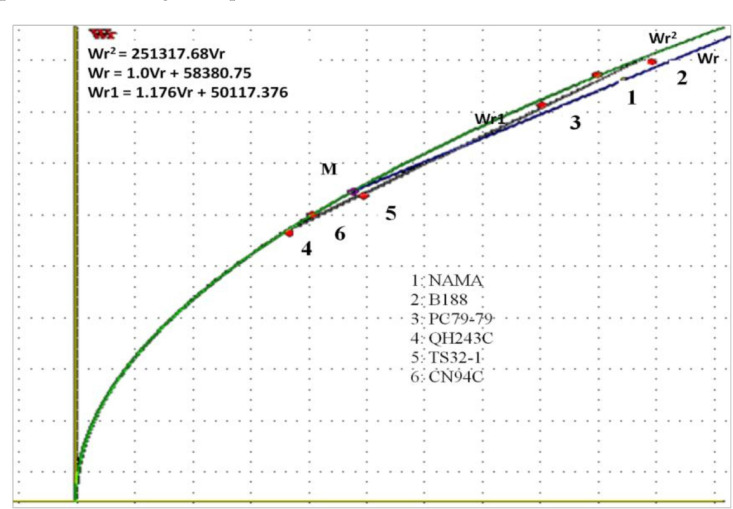
Covariance (Wr)/variance (Vr) graph for the AUDPC of groundnut early leaf spot disease. Wr: covariance between a parent r and its progeny; Vr: variance between a parent r and its progeny; Wr1: regression line; Wr2: parabola; Wr: tangent to parabola.

A Hayman graphical analysis was conducted to assess the genetic relationships among the parents. It provides a measure of the adequacy of the model used, average dominance, and distribution of dominant and recessive genes. The position of the regression line on the Vr–Wr graph provides information about the average degree of dominance. Figures 1 and 2 show the Vr–Wr graph for the ELS severity score and AUDPC, respectively. The regression line of the two traits passes above the origin. The coefficients of regression of Vr on Wr were 1.01 for both traits and did not differ from unity. All the parents tended to cluster along the regression line for the two traits, except parents NAMA and QH243C, for the ELS severity score. In addition, parents QH243C, TS32-1, and CN94C were clustered closer to the origin of the regression line, whereas the parents NAMA, PC79-79s and B188 were in the middle and end of the regression line.

## 4. Discussion

The six parental lines and their 30 F2 progenies showed highly significant differences for the ELS severity score and AUDPC. These differences were due to the considerable genetic diversity among the materials tested. High variability among groundnut genotypes in terms of leaf spot disease resistance, including ELS, were also previously reported [1,25–27].

The Griffing analysis of variance indicated a highly significant GCA for ELS severity and AUDPC, indicating the role of additive gene action in the control of the two traits. On the other hand, the SCA was significant for the ELS severity score only. The results suggest that both additive and non-additive gene effects are involved in the expression of ELS disease resistance in groundnut. Similar findings of additive and non-additive gene actions for yield-related traits and diseases, including ELS resistance, were reported earlier [1,26–29]. In addition, the reciprocal effect was highly significant for the two traits, indicating the contribution of cytoplasm and nuclear genes effects to ELS resistance. The estimates of the genetic components indicated that variance due to GCA was higher in magnitude compared to variance due to SCA for the two traits. Their ratio was greater than unity, indicating the profound influence of additive gene action in the control of ELS disease in groundnut. This is a desirable phenomenon necessary for making progress in breeding for ELS resistance. These findings are in accordance with previous results [1,26,27,30].

The general combining ability indicates the average performance of a line in cross combination [22]. In general, desirable parents are those with significant GCA effects in the center direction for the trait of interest [31]. In this study, negative GCA effects were desirable, since they indicate a contribution to resistance, while positive values indicate a contribution to susceptibility. The NAMA parents, followed by PC79-79 and B188 recorded negative GCA, suggesting that the use of these parents in breeding for resistance to ELS resistance would be expected to produce progenies with increased resistance. In addition, these three parents showed low mean disease scores. Some crosses involving these resistant parents exhibited negative SCA effects. For the SCA effects, 10 of 30 possible combinations exhibited negative SCA effects for ELS resistance components, indicating that the resistance of these progenies was higher than would be expected from the average of their parents. For the ELS severity score, five cross combinations, including NAMA × B188, PC79-79 × TS32-1, PC79-79 × CN94C, QH243C × TS32-1, and QH243C × CN94C, showed negative SCA effects. For AUDPC, negative SCA effects were observed for nine combinations (NAMA × PC79-79, NAMA × B188, NAMA × CN94C, B188 × PC79-79, B188 × QH243C, PC79-79 × TS32-1, QH243C × TS32-1, QH243C × CN94C, and TS32-1 × CN94C). Four cross combinations (NAMA × B188, PC79-79 × TS32-1, PC79-79 × CN94C, and QH243C × TS32-1) showed negative SCA effects for both ELS severity score and AUDPC. These crosses could be considered the best cross combinations for ELS resistance.

Hayman’s analysis revealed that both the additive “a” and dominance “b” genetic components were significant, indicating their importance in the regulation of ELS resistance in groundnut. The results were similar to Griffing’s analysis. Additionally, the mean dominance effect “b1”, additional dominance effect due to the parents “b2”, and residual dominance effect “b3” were significant for both the ELS severity score and AUDPC. The significance of “b1” indicated that the dominance was unidirectional, while the significance of “b2” indicated an asymmetry of gene distribution. Thus, susceptible parents (QH243C, TS32-1, and CN94C) harbored considerably higher numbers of dominant genes than the resistant parents NAMA, PC79-79, and B188. The presence of specific dominance in some crosses (NAMA × B188, PC79-79 × TS32-1, QH243C × TS32-1, PC79-79 × CN94C, and QH243C × CN94C) was confirmed by the significance of “b3” for the ELS severity score. Similar results were earlier reported for rust [25] and for leaf spot diseases of groundnut, including ELS [15,26].

The results also revealed a predominance of additive variance (D) compared to the components associated with dominance variance (H^1^ and H^2^) for the ELS severity score and AUDPC. These indicate that resistance to ELS is predominantly regulated by additive gene action. The findings are in harmony with the results of Griffing’s analysis. Considering the dominance components, the magnitude of H^1^ was greater than H^2^, indicating the non equality of dominant and recessive alleles in all loci controlling ELS resistance. For both the ELS severity score and AUDPC, positive F item values were observed, which measured the covariance of the additive and dominance effects, indicating the predominance of dominant alleles in the parents. The high F value also indicated that positive and negative alleles were not equal in proportion. The proportion of positive alleles was greater than that of the negative alleles in the parents at any locus. The ratio of (H^1^/D)^1/2^, which measures the average degree of dominance, and the ratio of the total number of dominant to recessive genes in all parents (kd/(kd + kr) were less than unity, suggesting partial dominance and confirming the presence of recessive alleles for ELS resistance in all parents. Heritability estimates showed high (>60%) broad sense heritability and narrow heritability for the two related traits studied, indicating that ELS resistance is highly heritable. Similar results were reported for ELS and late leaf spot [32–34].

The Vr–Wr graph showed a coefficient of regression (1.01) not significantly different from unity for the two traits, suggesting the absence of epistasis, and rather additive and dominance gene action in ELS expression. An average partial dominance might be considered, since the regression line crossed the Wr axis above the origin. These results are in accordance with those reported for yield and yield-related traits [1]. Considering the distribution of array points along with the regression line, the parents QH243C, TS32-1, and CN94C were clustered closer to the origin of the regression line, suggesting that they contain larger proportions of dominant alleles. However, the resistant parents NAMA, PC79-79, and B188 were farthest from the origin and closer to the middle of the regression line, suggesting that they contained equal proportions of dominant and recessive alleles.

In summary, based on both the GCA and SCA effects, the study has identified suitable parents that can be used for an ELS resistance breeding program in West and central Africa. In addition, the study has revealed the existence of additive and dominance gene action for controlling ELS disease resistance with a predominance of additive gene action. This suggests that selection for the two ELS resistance traits could be easily practiced in early generations to develop ELS-resistant lines. Effective early generation selection for resistance would be advantageous and allow for procedures such as independent culling, tandem selection, or index selection involving other traits, such as yield, seed quality, and multiple pest resistance [26]. Earlier studies on early generation selection for yield and ELS indicated a selection advantage among crosses compared to individual plant selection or within-family selection [35,36]. However, the positive correlation of ELS resistance traits with yield and its components reported elsewhere [12,32,34,37,38] requires developing large number of crosses to break the undesirable association, and identify high-yielding genotypes with acceptable ELS resistance.

## 5. Conclusions

The study, using populations developed from varieties in West and Central Africa, was useful to understanding the mode of inheritance, identifying good combiners, and evaluating the breeding potential of populations for ELS resistance. The results indicated more recessive alleles in the resistant parents, confirming the recessive inheritance of ELS. Both additive and dominance gene actions control ELS disease resistance, with a predominance of additive gene action. The results suggest that selection of appropriate parents for breeding programs for ELS resistance should be based on both GCA and SCA effects. NAMA, PC79-79, and B188 are good combiners for use in breeding for ELS resistance in the region. The best crosses, including NAMA × PC79-79, B188 × PC79-79, B188 × QH243C, and NAMA × CN94C, were identified for further evaluation.
